# Profiling Tumor Immune Microenvironment of Non-Small Cell Lung Cancer Using Multiplex Immunofluorescence

**DOI:** 10.3389/fimmu.2021.750046

**Published:** 2021-11-04

**Authors:** Haoxin Peng, Xiangrong Wu, Ran Zhong, Tao Yu, Xiuyu Cai, Jun Liu, Yaokai Wen, Yiyuan Ao, Jiana Chen, Yutian Li, Miao He, Caichen Li, Hongbo Zheng, Yanhui Chen, Zhenkui Pan, Jianxing He, Wenhua Liang

**Affiliations:** ^1^ Department of Thoracic Oncology and Surgery, China State Key Laboratory of Respiratory Disease and National Clinical Research Center for Respiratory Disease, The First Affiliated Hospital of Guangzhou Medical University, Guangzhou, China; ^2^ Nanshan School, Guangzhou Medical University, Guangzhou, China; ^3^ Genecast Biotechnology Co., Ltd., Beijing, China; ^4^ Department of General Internal Medicine, Sun Yat-sen University Cancer Centre, State Key Laboratory of Oncology in South China, Collaborative Innovation Centre for Cancer Medicine, Guangzhou, China; ^5^ School of Medicine, Tongji University, Shanghai, China; ^6^ Department of Medical Oncology, Shanghai Pulmonary Hospital, Tongji University Medical School Cancer Institute, Tongji University School of Medicine, Shanghai, China; ^7^ Institute of Infectious Diseases, Beijing Ditan Hospital, Capital Medical University, Beijing Key Laboratory of Emerging Infectious Diseases, Beijing, China; ^8^ Department of Oncology, Qingdao Municipal Hospital, Qingdao, China; ^9^ Medical Oncology, The First People’s Hospital of Zhaoqing, Zhaoqing, China

**Keywords:** tumor immune microenvironment, immune landscape, immune subtyping, multiplex immunofluorescence, immune-related risk score

## Abstract

This study attempted to profile the tumor immune microenvironment (TIME) of non-small cell lung cancer (NSCLC) by multiplex immunofluorescence of 681 NSCLC cases. The number, density, and proportion of 26 types of immune cells in tumor nest and tumor stroma were evaluated, revealing some close interactions particularly between intrastromal neutrophils and intratumoral regulatory T cells (Treg) (*r* ^2^ = 0.439, *P* < 0.001), intrastromal CD4+CD38+ T cells and CD20-positive B cells (*r* ^2^ = 0.539, *P* < 0.001), and intratumoral CD8-positive T cells and M2 macrophages expressing PD-L1 (*r* ^2^ = 0.339, *P* < 0.001). Three immune subtypes correlated with distinct immune characteristics were identified using the unsupervised consensus clustering approach. The immune-activated subtype had the longest disease-free survival (DFS) and demonstrated the highest infiltration of CD4-positive T cells, CD8-positive T cells, and CD20-positive B cells. The immune-defected subtype was rich in cancer stem cells and macrophages, and these patients had the worst prognosis. The immune-exempted subtype had the highest levels of neutrophils and Tregs. Intratumoral CD68-positive macrophages, M1 macrophages, and intrastromal CD4+ cells, CD4+FOXP3- cells, CD8+ cells, and PD-L1+ cells were further found to be the most robust prognostic biomarkers for DFS, which were used to construct and validate the immune-related risk score for risk stratification (high *vs*. median *vs*. low) and the prediction of 5-year DFS rates (23.2% *vs*. 37.9% *vs*. 43.1%, *P* < 0.001). In conclusion, the intricate and intrinsic structure of TIME in NSCLC was demonstrated, showing potency in subtyping and prognostication.

## Introduction

According to the global cancer statistics reported by the International Agency for Research on Cancer, lung cancer (LC) is the second most commonly diagnosed cancer and the most common cause of cancer death worldwide (1). Non-small cell lung cancer (NSCLC) accounts for around 85% of LC, and it encompasses two major histological subtypes: lung adenocarcinoma and squamous cell lung cancer, respectively (2). Despite the immense improvements in new drugs and systemic therapy, the 5-year overall survival (OS) rate for advanced NSCLC patients was less than 5% (3).

Emerging evidence shows that the tumor immune microenvironment (TIME) is the key determinant of LC development and the prognosis of patients (4, 5). The TIME mainly contains neoplastic cells, stromal cells, and diverse immune cells, and these components interact mutually through complex cellular and molecular mechanisms, which influence tumor progression, metastasis, and clinical outcomes like treatment tolerance (6). The location, type, density, and functional state of immune cells constitute the immune contexture of TIME, varying in patients with NSCLC. The immune cells may have dual impacts for both anti-tumor and pro-tumor effects—for instance, CD8+ T cells and natural killer (NK) cells mediate antitumoral responses, demonstrating a better OS, disease-free survival (DFS), and progression-free survival. On the contrary, the regulatory T cells (Tregs) can secrete inhibitory cytokines, such as transforming growth factor (TGF)-β and interleukin (IL)-10, contributing to LC progression *via* angiogenesis and immunosuppression through inhibition of the anti-tumor effect of T-helper (Th1) cells as well as attracting activated Th2 cells (7–10).

Immunotherapy, mainly enhancing the anti-tumor immune responses through targeting the T cell regulatory pathway in TIME, has shown enormous potential and promising results for improving disease control in NSCLC patients in recent years (11)—for instance, the 5-year OS rate triggered by immunotherapy, especially immune checkpoint blockades (ICB), now surpasses 25% for patients with high programmed cell death protein ligand-1 (PD-L1) expression (tumor proportion score ≥50%) (12), but the long-term clinical benefits occur only in a limited portion of patients (13). Current studies pointed that PD-L1, tumor mutational burden (TMB), and intratumoral heterogeneity may provide hints of prognosis with immunotherapy in NSCLC—for instance, a high TMB (>10 mutations/megabase) can select patients with NSCLC who may benefit from ICB, irrespective of the expression levels of PD-L1. Moreover, recent studies implied that the combination of multiple biomarkers may better predict ICB response. McGrail et al. reported that, in lung and bladder cancers where CD8 T cell counts positively associated with neoantigen load, high-TMB tumors demonstrated significantly higher objective response rates than low-TMB tumors, while this trend was not observed in breast and prostate cancers which show no correlation between CD8 T cell counts and neoantigen load (14). Additionally, high somatic copy number alteration burden was associated with low infiltration levels of NK cells and CD8 T cells and poor response of ICB (15). However, there is no consensus regarding the best predictive biomarker of prognosis (16). Still we know little about the TIME of NSCLC and how this information could be utilized to design appropriate therapies for distinct patient subgroups. Hence, a vital unmet need is to investigate the critical components and related cellular and molecular mechanisms responsible for immune responses, exhaustion, or ignorance to modify the TIME and design effective therapies.

Previous traditional immunohistochemistry (IHC)-based studies are usually limited to a relatively small sample size and few immune cell types, making it insufficient to exhibit the immune landscape of TIME fully. Recently, the multiplex immunofluorescence (MIF) approach has been demonstrated to provide a unique perspective into the spatial relationships among immune cells, stromal cells, and tumor cells within the complex TIME. The MIF also avoids the traditional shortcomings of IHC, such as low reproducibility and subjective scoring system (17, 18).

In this study, we described the immune landscape of NSCLC *in situ* and identified a novel stratification of TIME by three immune subtypes using MIF. We also established the immune-related risk score (IRRS) model as a robust prognostic biomarker for DFS.

## Materials and Methods

### Patient Cohort

From 2009 to 2011, we collected a consecutive series of 681 NSCLC patients, from stage I to III, who had undergone lobectomy/sub-lobectomy and lymph node dissection at the First Affiliated Hospital of Guangzhou Medical University. Written informed consent was obtained from all patients, permitting the MIF analyses of the biological samples (19).

The inclusive criteria were as follows: (1): single primary NSCLC, (2) stage I to III, (3) underwent anatomical resection in combination with lymphadenectomy (systematic lymph node sampling or systematic lymph node dissection) according to the National Comprehensive Cancer Network criteria (19, 20), (4) all resected tissues and lymph nodes were confirmed by pathology, and (5) sufficient resected tissues for MIF test. Patients were excluded if any of the following criteria were met: (1) multiple LC, (2) small cell lung cancer or non-invasive LC like lung adenocarcinoma (LUAD) *in situ* and minimally invasive LUAD, (3) diagnostic biopsy in pre-operation, and (4) preoperative neoadjuvant therapy.

### Multiplex Immunofluorescence Detection

MIF staining was conducted at Genecast Biotechnology Co., Ltd. (Beijing, China). Briefly, a 4-μm-thick section was cut from formalin-fixed paraffin-embedded lung cancer tissues for each panel detection. The slides were deparaffinized, rehydrated, and subjected to epitope retrieval by boiling in Tris-EDTA buffer (pH = 9; Klinipath #643901, Duiven, Netherlands) for 20 mins at 97°C. Endogenous peroxidase was then blocked by incubation in Antibody Diluent/Block (PerkinElmer #72424205, Massachusetts, USA) for 10 mins, and protein was subsequently blocked in 0.05% Tween solution containing 0.3% bovine serum albumin for 30 mins at room temperature. Only one antigen was detected in each round, including primary antibody incubation, secondary antibody incubation, and tyramine signal amplification (TSA) visualization, followed by labeling the next antibody after epitope retrieval and protein blocking as before. CD4, CD20, CD38, CD66b, and FOXP3 for panel 1 and CD8, CD68, CD133, CD163, and PD-L1 for panel 2 were sequentially detected.

The primary antibodies for CD8 (ZA-0508, clone SP16, Zsbio, 1:100), CD20 (ab9475, abcam, 1:50, Zsbio, 1:100), CD38 (ZM0422, clone SPC32, Zsbio, 1:400), CD66b (ab214175, polyclonal antibody, abcam, 1:50), CD68 (ZM-0060, clone KP1, Zsbio, 1:100), CD163 (ZM-0428, clone 10D6), PD-L1 (13684s, clone E1L3N, CST, 1:100), and FOXP3 (ab20034, clone 236A/E7, abcam, 1:100) were incubated for 1 h at room temperature. Those for CD4 (ZM0418, clone UMAB64, Zsbio, 1:200) and CD133 (ab19898, polyclonal antibody, abcam, 1:400) were incubated overnight at 4°C.

Anti-rabbit/mouse horseradish peroxidase (Zsbio # PV-6002 or PV-8000) were used as the secondary antibody and incubated at 37°C for 10 mins. TSA visualization was then performed with the opal seven-color multiplex immunohistochemistry kit (NEL797B001KT, PerkinElmer, Massachusetts, USA), containing fluorophores (4′,6-diamidino-2-phenylindole, DAPI), Opal 520 (CD20 and CD163), Opal 540 (CD38), Opal 570 (PD-L1 and CD4), Opal 620 (CD8), Opal 650 (CD66b and CD133), Opal 690 (CD68 and FOXP3), and TSA Coumarin system (NEL703001KT, PerkinElmer, Massachusetts, USA). After labeling all of the five antigens for each panel, microwave treatment was performed to remove the TSA–antibody complex with Tris-EDTA buffer (pH = 9; Klinipath #643901, Duiven, Netherlands) for 20 mins at 97°C. All the slides were counterstained with DAPI for 5 mins and were enclosed in Antifade Mounting Medium (NobleRyder #I0052, Beijing, China) and prepared for imaging. Fresh whole-tissue-section cuts from normal human tonsils with both primary and secondary antibody incubation were included in each staining batch as the positive control, and the interexperimental reproducibility was assessed, while normal human tonsils with secondary antibody incubation but without primary antibody incubation were set as the negative control (**Supplementary Figure S1**).

The slides were scanned using the PerkinElmer Vectra (Vectra 3.0.5; PerkinElmer, Massachusetts, USA). Multispectral images were unmixed with spectral libraries built from single-stained tissue images for each antigen, using the inForm Advanced Image Analysis software (inForm 2.3.0; PerkinElmer, Massachusetts, USA).

For batch analysis, firstly, an experienced pathologist (Dr. Bai Xuejuan) sketched the distinct tumor nest (TN) and tumor stroma (TS) using 10 to 15 representative multispectral images in the inForm software to train the algorithm. Afterward, the inForm software can automatically detect and segment specific tissue types into TN and TS based on tissue morphologies using artificial intelligence-powered feature recognition algorithms. Cell segmentation was also conducted with the algorithm. The pathologist (Dr. Wang Xin) judged whether an antigen is expressed positively on a particular cell type by referring to the positive control (*i*.*e*., normal human tonsil tissue with both primary and secondary antibody incubation) and the negative control (*i*.*e*., normal human tonsil tissue with secondary antibody but without primary incubation) as mentioned above and then determined the appropriate positive threshold for each biomarker according to the signal intensity in the inForm software. Then, a superior pathologist, Dr. Bai Xuejuan, reviewed and judged the accuracy of the results by Dr. Wang Xin. Finally, disagreements were resolved by consensus between these two reviewers, and Dr. Bai Xuejuan determined the ultimate positive threshold. The inForm software can subsequently automatically determine the expression levels of different biomarkers across the slides using the same positive threshold set by the pathologist. We defined X, 2X, and 3X as the threshold of the signal intensity of low fluorescence intensity (+), median fluorescence intensity (++), and high fluorescence intensity (+++), respectively, and the “POS” (*i*.*e*., positive) value equals X + 2X + 3X. The density (*n*/mm^2^), number (*n*/sight), and percentage (%/sight) of immune markers in TN and TS were all calculated. The histochemistry score (H-score) was analyzed with the formula of H-score = (high fluorescence intensity)% × 3+ (median fluorescence intensity)% × 2+ (low fluorescence intensity)% × 1. In total, 26 kinds of immune cells, including 66 kinds of immune biomarkers, were test and calculated. Immune cell types represented by biomarkers were labeled through literature retrieval (21) (**Supplementary Table S1**). Cells with an expression of CD68 was identified as pan-macrophages, while CD68+CD163- was identified as M1 macrophages, and CD68+CD163+ was identified as M2 macrophages.

### Defining the Immune Landscape

The immune landscape of NSCLC was conducted with a MIF test for 681 cases, demonstrating the intricate and intrinsic structure of TIME and visualizing the immune features of individual patients.

### Discovery of the Immune Subtypes

Unsupervised consensus clustering is a class discovery approach to detect unknown possible clusters consisting of individual items with similar intrinsic features (22). Based on the proportion of 26 kinds of immune cells both in TN and TS, distinct subgroups of 681 samples were identified, during which 80% of the samples were extracted 100 times in turn, and a hierarchical clustering analysis was performed based on the Euclidean distance between data points. The consensus clustering results were subsequently tested using the cumulative distribution function plot corresponding to the consensus matrices. Then, the results of clustering were verified by employing principal component analysis (PCA).

### Evaluating the Cellular and Clinical Characteristics Correlated With the Immune Subtypes

Kruskal–Wallis (K–W) test and box plots were used to visualize the disparities of immune cell proportion among different clusters. The log-rank test was initially employed to evaluate the prognostic significance of immune subtypes. The multivariable Cox proportional hazards regression analysis was then used for further assessment with adjustments for age, sex, T stage, N stage, vascular cancer embolus, and number of lymph node resection, and DFS was considered as the endpoint. We also utilized the chi-square test to investigate the heterogeneity of the clinical characteristics in the three clusters.

### Profiling the Prognostic Value of Immune Biomarkers

Differences in the proportion of 26 kinds of immune cells among T stage, N stage, and clinical stage were analyzed through K-W test. Multivariable Cox regression with age, sex, histological types, T stage, and N stage as covariates was utilized to identify the prognostic value of 66 immune biomarkers. We further classified the values of immune biomarkers into high-value and low-value subtypes by the optimal cutoff point according to the built-in risk scoring formula in X-tile and assessed the differences in DFS.

### Construction and Validation of the Immune-Related Risk Score

The entire cohort (*n* = 681) was divided into the training cohort (*n* = 477) and the testing cohort (*n* = 204). Immune cells significantly associated with DFS through multivariable Cox regression (*P* < 0.05) in the entire cohort were selected as the candidate factors. The least absolute shrinkage and selection operator (LASSO) regression model was then used for profiling the most robust prognostic immune cells among the candidate factors, and the optimal lambda value was determined by 10-fold cross-validation (23). IRRS model was ultimately conducted by the regression coefficients originated from multivariable Cox regression method to multiply the proportion of immune cells in the training cohort:


$$\sum_{i=1}^{\text{n}}\text{ln}(\text{HR}i)*\text{proportion}i$$

in which HR*_i_* is the hazard ratio (HR), and proportion*_i_* is the proportion for the *i*-th immune cells. Multivariable Cox proportional hazards regression analysis with adjustments for sex, age, T stage, N stage, number of lymph node resection, and vascular cancer embolus was conducted to investigate the prognostic significance of IRRS in the training cohort. We further divided IRRS into high-IRRS, median-IRRS, and low-IRRS groups by the optimal truncation values to seek out the difference in DFS. The performance and robustness of IRRS in the training cohort was further tested in both the testing cohort and the entire cohort with the same formula and cut-off values.

### Statistical Analysis

Using R package psych (version 2.0.12), the Spearman rank correlation test was conducted to explore the correlations between immune cells in TN and TS, during which the correlation coefficients and their *P*-values were calculated, and the correlations were shown in dot–line charts based on R package ggpubr (version 0.4.0). The value of the coefficient ranges from -1 to 1, with 1 and -1 being the strongest positive and negative correlation, respectively. The absolute value of the correlation coefficient of “<0.30”, “0.30–0.50”, and “>0.50” was defined as weak, modest, and strong correlations in our study, respectively. R package ConsencusClusterPlus (version 1.54.0) was used to perform unsupervised consensus clustering analysis to explore the intricate relationships of the immune cells, and the clustering results were verified with PCA using the R package FactoMineR (version 2.4). Besides this, we explored the differences of the clinical characteristics among each cluster by percentage component bar chart and Sankey plot analysis, which were performed by R package ggplot2 (version 3.3.3) and ggalluvial (version 0.12.3), respectively. After that, in order to construct an IRRS-based prognostic model, we utilized LASSO regression using the glment package (version 4.0.2) in R software for high-dimensional data to select the most useful prognostic factors. The receiver operating characteristic (ROC) curve and time-dependent area under curve (AUC) were used to test the accuracy of the IRRS model using R package timeROC (version 0.4) (24). We also performed multi-variable Cox regression analysis to assess whether IRRS was independent of other clinical characteristics. The survival distribution of the DFS curves were estimated by Kaplan–Meier method, and the two-sided log-rank test as implemented in the R package survminer (version 0.4.8). All statistical analyses were performed using R software (version 4.0.3) and SPSS software (version 23.0). Multi-variable Cox regression analysis was used to determine whether the immune markers were independent of other clinical characteristics and significantly related to DFS. The HR, 95% confidence interval (CI), and *P*-value for each immune marker were calculated. Pearson’s chi-square test and Fisher’s exact test were applied for comparison between categorical variables. Non-parametric analysis (Mann–Whitney *U*-test or K–W test) was used for non-normally distributed rank/ordered variables and data, while continuous variables were analyzed by *T*-test. X-tile software was used to divide the values into several groups through the built-in risk scoring formula based on the combined model with the optimal cutoff points (25). All *P*-values were two-sided, and *P*-value <0.05 was considered statistically significant.

### Data Availability

There were no datasets generated or analyzed during the current study.

### Ethics Approval

This study obtained ethics approval from The First Affiliated Hospital of Guangzhou Medical University. The study was conducted following the Declaration of Helsinki (as revised in 2013) (26).

## Results

### Characteristics of the Patients

Six hundred and eighty-one patients met the criteria and were included in this study, the baseline characteristics of whom were presented in **Supplementary Table S2**. Of the included cases, 321 (47.1%) were more than 60 years old, and 398 (58.4%) were male. LUAD was the dominant histological subtype (479, 70.5%). Clinical stages IA, IB, IIA, IIB, IIIA, and IIIB accounted for 22.0%, 31.1%, 16.2%, 5.5%, 24.8%, and 0.5%, respectively.

### Immune Landscape and Interactions Among Immune Cells

**Figure 1:**
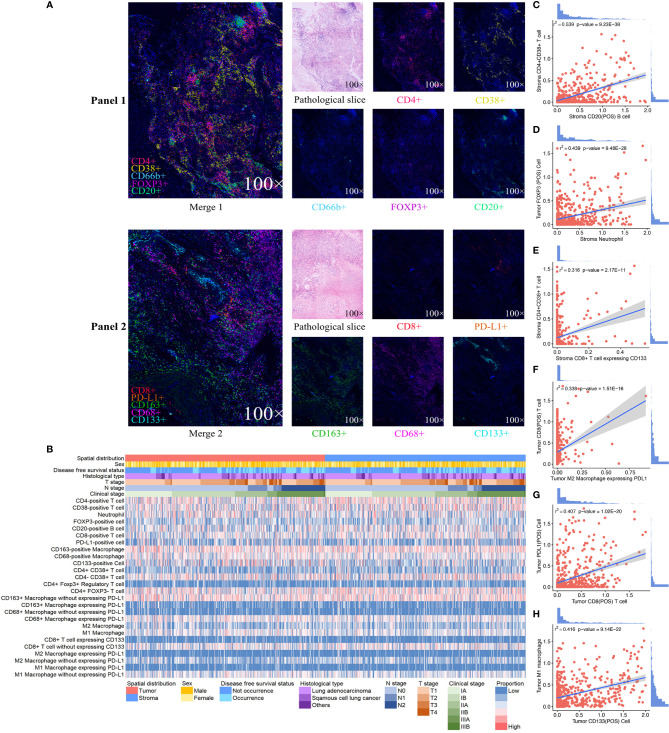
Expression profile of 10 immune biomarkers in non-small cell lung cancer. Single and merged immunofluorescence images and pathological slices are shown accordingly **(A)**. Immune landscape of the tumor immune microenvironment (TIME) illustrates the log percentage (lg%) of each type of immune cell within tumor nest and tumor stroma. Each value corresponds to the clinical characteristics of the patients, including sex, disease-free survival status, histological type, clinical stage, T stage, and N stage **(B)**. The dotted line graphs illustrate the correlations between immune cells in TIME, and the bar graph shows the distribution of the logarithmic percentage (lg%) of the proportion: **(C)** intrastromal CD20-positive B cells and intrastromal CD4+CD38+T cells, **(D)** intrastromal neutrophils and intratumoral FOXP3-positive cells, **(E)** intratumoral CD8-positive T cells and M2 macrophages expressing PD-L1, **(F)** intratumoral CD38-positive T cells and intratumoral CD20-positive B cells, **(G)** intratumoral CD8-positive T cells and intratumoral PD-L1-positive cells, and **(H)** intratumoral CD133-positive cells and intratumoral M1 macrophages.

**Figure 2:**
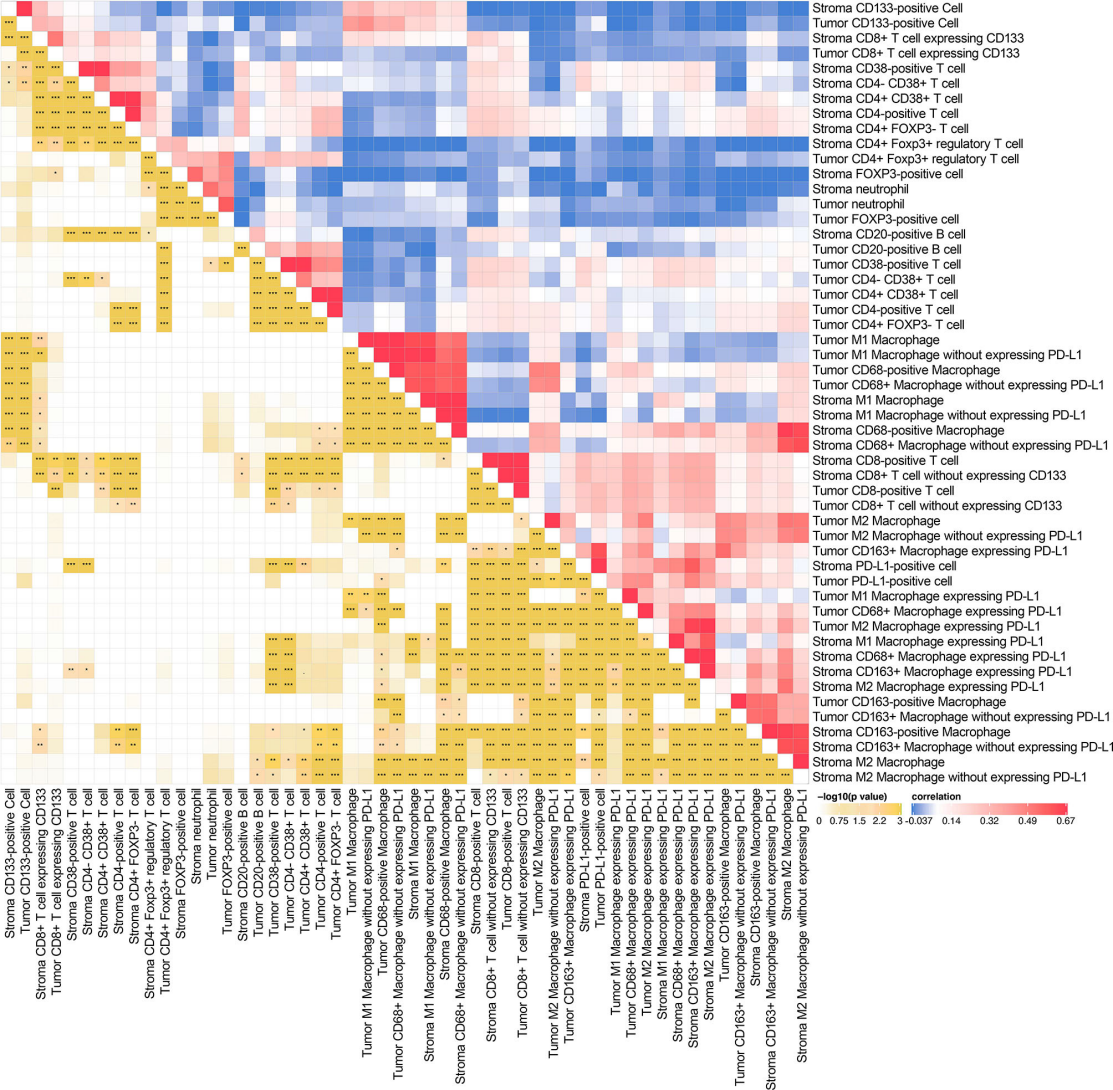
Spearman’s rank correlation matrix (right half) and corresponding *p*-value (left half) among various intratumoral and intrastromal immune cell types. *P < 0.05, **P < 0.01, ***P < 0.001.

We exhibited the representative MIF images of each immune biomarker in TN and TS (**Figure 1A**). The immune landscape of NSCLC, both in TN and TS, was demonstrated (**Figure 1B**). Strong interactions were observed particularly on intrastromal CD20-positive B cell and intrastromal CD4+CD38+ T cell (*r* ^2^ = 0.539, *P* = 9.23E-38), neutrophil and FOXP3-positive cell (*r* ^2^ = 0.501, *P* = 5.17E-32 in TS and *r* ^2^ = 0.552, *P* = 7.00E-40 in TN, respectively) as well as intrastromal neutrophil and intratumoral Treg (*r* ^2^ = 0.439, *P* = 9.48E-28), intratumoral CD38-positive T cell and intratumoral CD20-positive B cell (*r* ^2^ = 0.525, *P* = 1.37E-35), intratumoral CD8-positive T cell and intratumoral M2 macrophage expressing PD-L1 (*r* ^2^ = 0.339, *P* = 1.51E-16), intratumoral PD-L1-positive cell and intratumoral CD8-positive T cell (*r* ^2^ = 0.407, *P* = 1.02E-20), and so on (**Figures 1C–H**, **2** and **Supplementary Tables S3**, **S4**). Moreover, modest correlations were also found between intratumoral CD133-positive cell and intratumoral M1 macrophage (*r* ^2^ = 0.416, *P* = 9.14E-22) and M1 macrophage without expressing PD-L1 (*r* ^2^ = 0.451, *P* = 2.25E-29), more specifically. Given that CD133 is usually defined as a marker of cancer stem cell (CSC) of LC, the interaction between CSC and macrophage may contribute to the mechanisms underlying immune escape (27–30). Previous studies have deemed CD38 as the marker of activated CD4+ T cell and CD133 as the marker of CD8+ T cell stemness, and a moderate correlation between CD4+CD38+ T cell and CD8+CD133+ T cell was observed (*r* ^2^ = 0.316, *P* = 1.89E-14) in TS rather than in TN (31–34). In addition, moderate correlations for intratumoral CD68+PD-L1+ macrophage and intratumoral CD8+ T cell (*r* ^2^ = 0.365, *P* = 5.82E-16) and intratumoral M2 macrophage expressing PD-L1 and intratumoral CD8+ T cell (*r* ^2^ = 0.339, *P* = 1.51E-16) were also presented, implying that the intratumoral macrophage may play a role in mediating exhausted CD8-specific immune response (35). As for the neutrophil and FOXP3-positive cell, previous studies have reported that tumor-associated neutrophil can recruit FOXP3-positive cells through chemokine ligand 2 (CCL)–chemokine receptor-2 (CCR) and CCL17–CCR4 pathways to form immunosuppressive TIME and promote tumor progression (36). It is noteworthy that the correlations among biomarkers within one district were assessed based on the quantitative expression of biomarkers rather than the spatial correlation analysis and, hence, may not be precise enough. Nevertheless, our findings implied the complicated associations among diverse biomarkers in the TIME, and future experimental studies as well as studies combined with quantitative and spatial analysis are necessary to further investigate the specific signaling pathways and the downstream immune responses (*e*.*g*., the secretion of chemokines). Moreover, we observed that the infiltrating levels of several cell types (neutrophils, CD68-positive macrophages, CD133-positive cells, M1 macrophages, CD68+PDL1+ macrophages, and M2 macrophages without expressing PD-L1) were higher in TN than in TS (**Supplementary Figure S2**).

### Components and Clinical Features of Immune Subtypes

**Figure 3:**
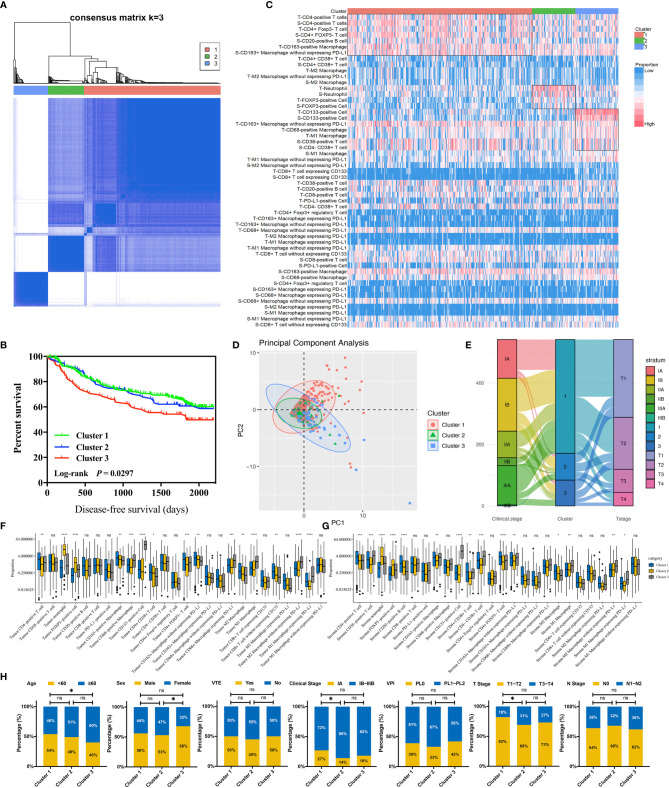
Identification and analysis of three immune clusters of 681 non-small cell lung cancer cases. **(A)** Consensus clustering matrix for *k* = 3. **(B)** Kaplan–Meier curve demonstrates the disease-free survival differences among three clusters. **(C)** The immune landscape of three clusters illustrates distinct cellular characteristics. **(D)** Principal component analysis of three clusters. **(E)** Sankey plot indicates the clinical characteristics differences among three clusters. **(F)** Infiltration disparities of three clusters in the tumor nest. **(G)** Infiltration disparities of three clusters in the tumor stroma. **(H)** Chi-square test reveals the disparities of the clinical characteristics of the patients among three clusters. *P < 0.05; **P < 0.01; ***P < 0.001; ****P < 0.0001; ns, nonsignificant; VTE, vascular tumor emboli; VPI, visceral pleural invasion.

Through conducting an unsupervised consensus clustering approach of 681 NSCLC cases, including cluster-consensus and item-consensus analyses, we identified three distinct immune subtypes based on the proportion of immune cells in TIME (**Figure 3A**). Kaplan–Meier survival analysis suggested significant differences in DFS among three immune subtypes (*P* = 0.0297). Subtype 1 had the longest DFS, while subtype 3 showed the worst (HR 1.50, 95%CI 1.07–2.11, *P* = 0.019) (**Figure 3B**), which were further supported by multivariable Cox regression analysis (HR 1.51, 95%CI 1.05–2.17, *P* = 0.026). We also observed a trend for longer DFS in immune class 2 (HR 0.76, 95%CI 0.48–1.20, *P* = 0.237) compared with class 3. Patients in immune subtype 1 had a marginally better DFS than immune subtype 2 (*P* = 0.471) (**Supplementary Table S5**).

**Figure 4:**
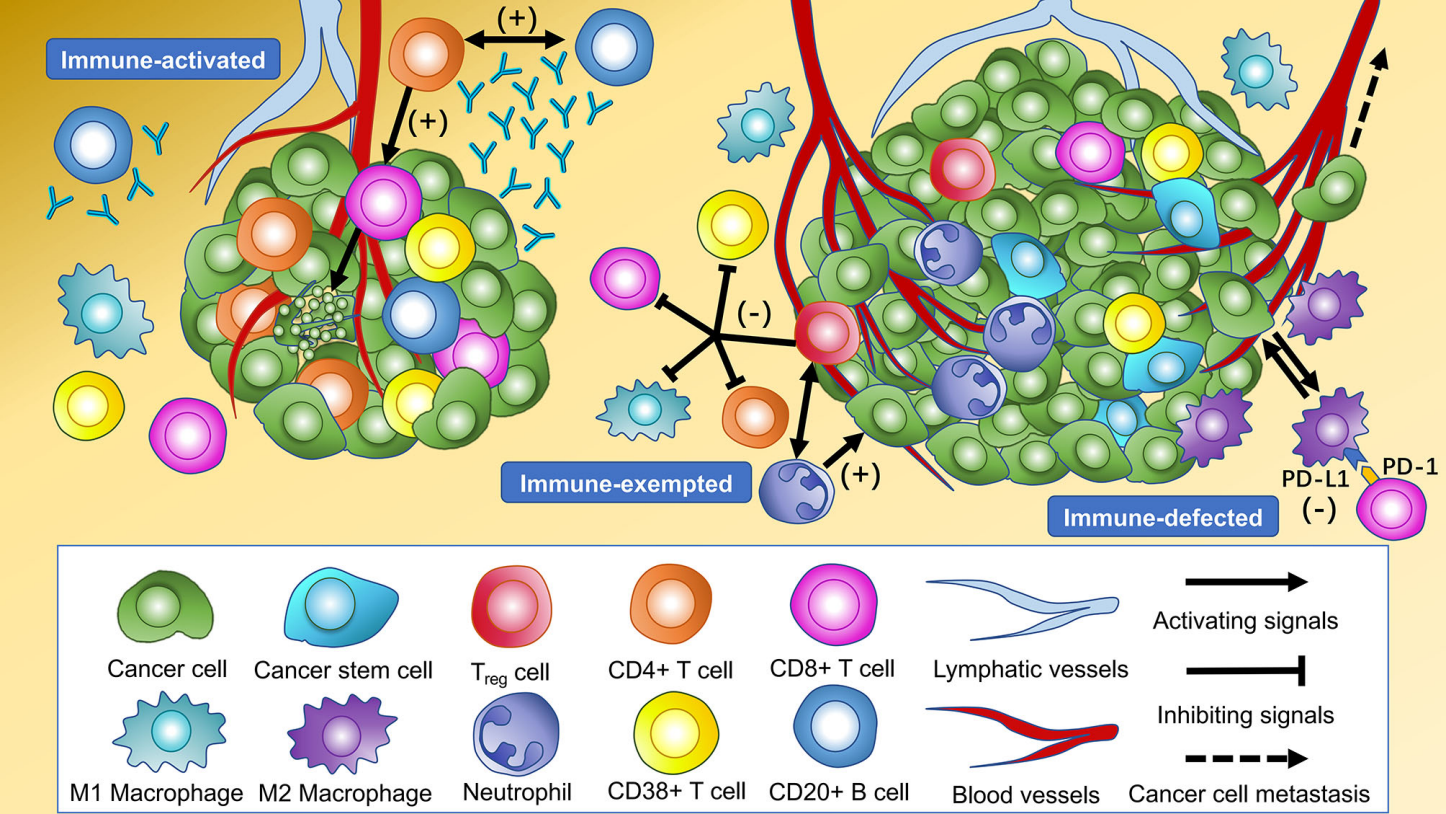
Cellular features of immune subtypes in the tumor immune microenvironment of non-small cell lung cancer (NSCLC). The immune-activated subtype is characterized by the highest levels of intratumoral and intrastromal CD4+ T cells, intrastromal CD20+ B cells, and intratumoral CD8+ T cells. CD20+ B cells present tumor antigens for activating CD4+ cells, and CD20+ B cells proliferate and differentiate into plasma cells to generate antibodies for antineoplastic effects with the help of cytokines secreted by CD4+ cells. The CD8+ T cells activated by CD4+ cells tend to kill the cancer cells in the tumor core directly. The highest levels of intratumoral and intrastromal regulatory T cells (Tregs) and neutrophils were observed in the immune-exempted subtype. Tregs produce immunosuppressive molecules which inhibit the activation and function of CD4+ T cells, CD8+ T cells, CD38+ T cells, and M1 macrophages to disrupt immune surveillance and promote tumor progression. Tregs may also recruit neutrophils through the chemokine ligand–chemokine receptor pathway. The immune-defected subtype has the highest levels of intratumoral and intrastromal cancer stem cells (CSC) and intratumoral macrophages. The macrophages in the immune-defected subtype are educated by the CSC to obtain pro-tumorigenic functions like angiogenesis and induce the exhaustion of anti-tumor cells. The immune-exempted and immune-defected subtypes are associated with a more advanced-stage NSCLC than the immune-activated subtype.

The distinct cellular features among three immune subtypes were shown (**Figures 3C–G**). Immune subtype 1 accounted for 68.2% of enrolled patients, in which the highest infiltration of CD4-positive T cells and CD20-positive B cells were observed and the proportion of both cells were higher in TS than in TN (*P* < 0.001). More specifically, CD4+CD38+ T cells and CD4+FOXP3- T cells were the major subsets of CD4-positive T cells rather than CD4+FOXP3+ Tregs. Besides this, the highest infiltrating levels of CD8-positive T cells were found in TN rather than in TS, indicating active anti-tumor immunity. It has been reported that B cells organized in tertiary lymphoid structures (TLS) could present tumor antigens for activating CD4-positive cells, and B cells could also proliferate and differentiate into plasma cells for generating antibodies for antineoplastic effects with the help of IL-4 secreted by CD4-positive cells (37). Hence, CD4-positive T cells and CD20-positive B cells possibly act as the “guides” in participating anti-tumor responses indirectly in TS through secreting IFN-γ and recruiting and activating T cells, B cells, and NK cells, while CD8-positive T cells tend to directly kill tumor cells in TN. Thus, this subtype was assumed to be “immune-activated” (**Figure 4**). Moreover, we also found a relatively high infiltration of M2 macrophages in this subtype. Considering that M2 macrophage was usually associated with pro-tumor effects like angiogenesis and immunosuppression, it suggested the existence of intracluster heterogeneity (38). Further investigation for the functional state showed that the majority was M2 macrophages without expressing PD-L1 rather than expressing PD-L1, implying that the immunosuppressive function had not yet been developed and the anti-tumor effects might still be dominant, which was consistent with the recent report (39). Immune subtype 3 accounted for 16.0% of the included patients, similar to immune subtype 2 (15.8%), which was characterized by the highest proportion of CD133-positive cells, M1 macrophages expressing PD-L1, and M2 macrophages without expressing PD-L1. CD133-positive cell, mainly CSC, could show unlimited capacity for self-renewal, which plays a vital role in inducing tumor recurrence, metastasis, and heterogeneous tumor cells (40). Previous studies have demonstrated an intimate connection between CSC and macrophage—for instance, CSC can recruit Tregs into TIME, which subsequently secret IL-10, and TGF-β in mediating an immunosuppressive microenvironment and induce macrophages to polarize into M2 subset, also known as the tumor-associated macrophages (TAMs) (41). The TAMs would also, in turn, impact CSC, like inducing the epithelial–mesenchymal transition of CSC to promote tumor invasion (42). Consequently, macrophages can be “educated” to develop pro-tumor effects under the impact of CSC, and so this subtype was regarded as “immune-defected” (**Figure 4**). Patients in immune-defected subtype were also associated with older age and with a higher proportion of male individuals (**Figure 3H**). The highest infiltration of neutrophils and FOXP3-positive cells in TN and TS was found in immune subtype 2, and as mentioned above, we also observed a strong correlation between neutrophils and FOXP3-positive cells. FOXP3-positive cells, mainly Tregs, are generally thought to disrupt anti-tumor immunity (43). Nevertheless, the prognostic effects of neutrophils in NSCLC are still conflicting to date. In general, high levels of N1 neutrophils showed superior outcomes, while N2 mainly indicated the negative, possibly through releasing matrix metallopeptidases-9 and elastase to drive the metastasis of LC cells (44, 45). Moreover, the levels of other infiltrating immune cells were the lowest in this subtype, and the T stage and clinical stage were more advanced (*P* < 0.05) (**Figure 3H**). Therefore, the formation of an immunosuppressive microenvironment and lack of immune responses made the cancer cells have the privileges and immunities from immune attack and so were regarded as “immune-exempted” (**Figure 4**) (46).

### Prognoses of Immune Biomarkers

Twenty-eight out of 66 kinds of immune biomarkers were significantly associated with DFS. Intrastromal CD4-positive T cell was manifested as an independent protective biomarker in DFS. CD4++ T cells showed a stronger protective effect towards DFS than CD4+ T cells and CD4+++T cells. Moreover, intrastromal CD4+FOXP3+ Tregs were significantly associated with a longer DFS. CD8++T cells indicated the strongest protective effect towards DFS compared with CD8+T cells and CD8+++ T cells. Intratumoral CD8-positive T cells expressing CD133 exhibited the strongest protective effect among all biomarkers (HR = 0.50, 95%CI 0.28–0.90). Intrastromal neutrophils and CD20-positive B cells were related to the tendency of a longer DFS.

**Figure 5:**
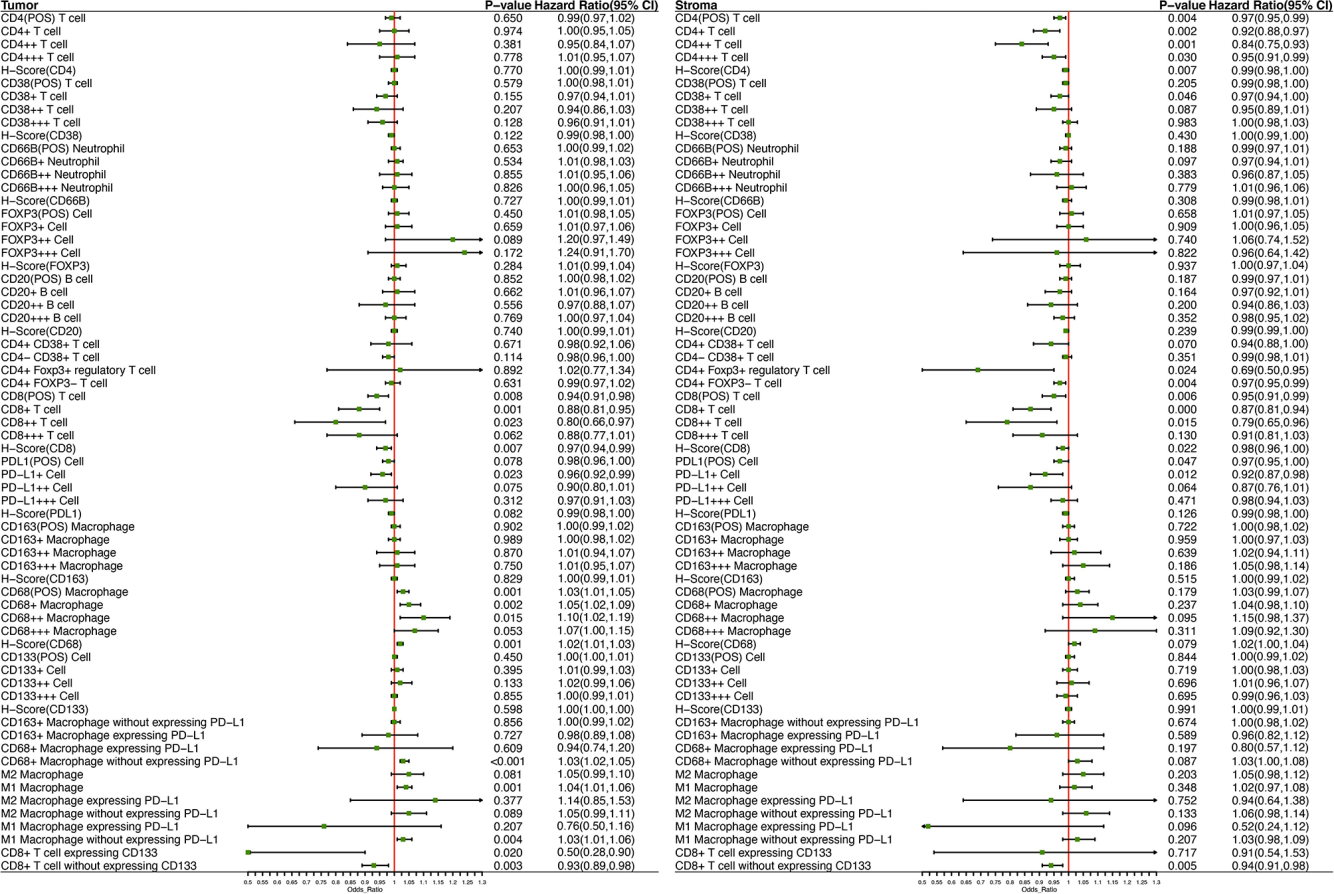
Forest plot demonstrates the prognostic significance of diverse immune biomarkers in the tumor nest and tumor stroma as implemented in the multivariable Cox analysis with age, sex, T stage, N stage, vascular cancer embolus, and the number of lymph node resections as covariates.

**Figure 6:**
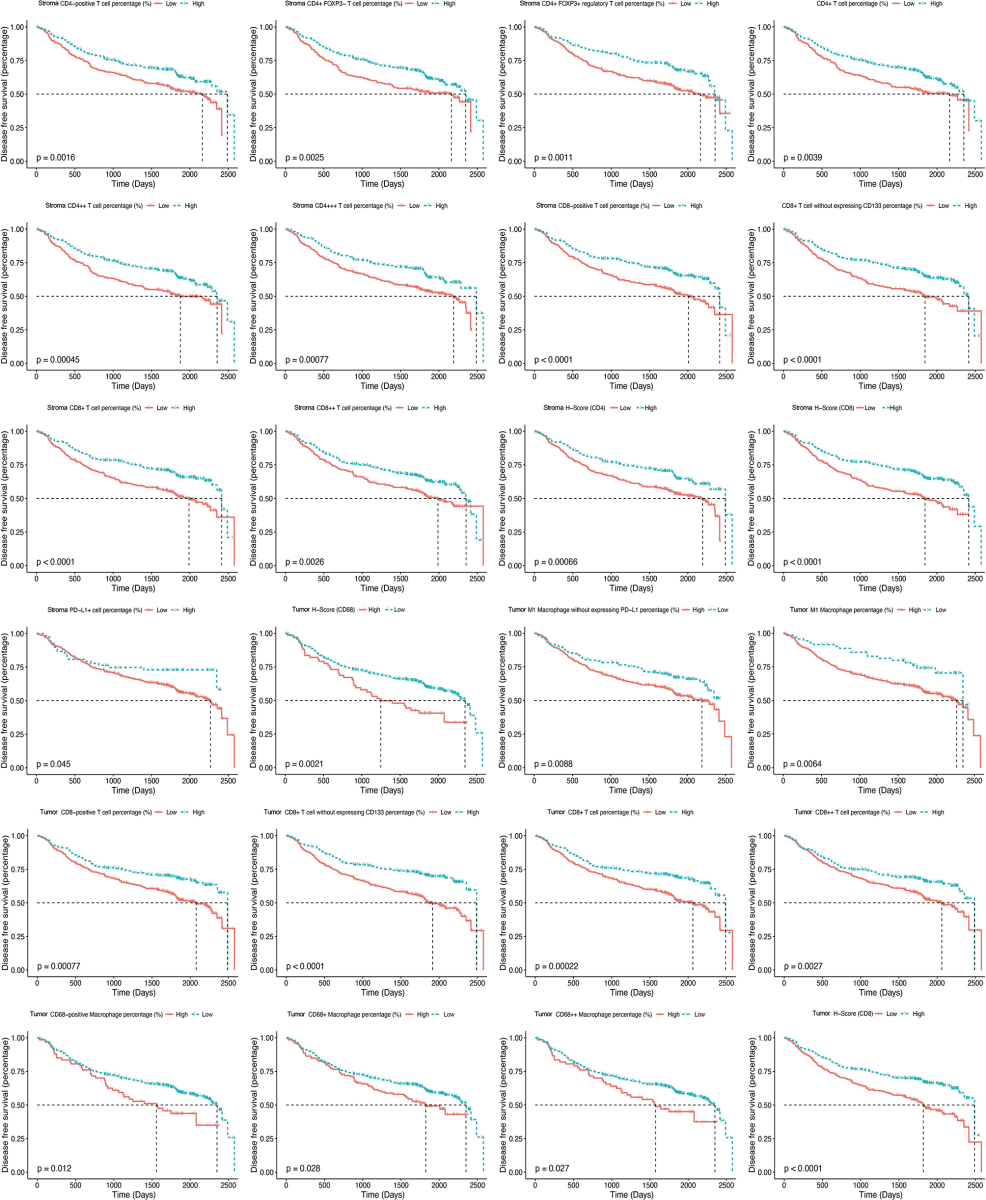
Kaplan–Meier curves illustrate the associations between the expression levels of immune biomarkers (high *vs*. low) within the tumor nest and tumor stroma and the disease-free survival of non-small cell lung cancer.

A higher infiltration of macrophages was observed to be associated with a worse outcome, and such effect was found to be more significant with the growth of fluorescence intensity. Further analysis revealed that both intrastromal and intratumoral CD68-positive macrophages expressing PD-L1 were associated with improved DFS, while CD68-positive macrophages without expressing PD-L1 were correlated with a worse prognosis (**Figure 5**). Moreover, a higher H-score (CD8) indicated a better DFS, whereas a higher H-score (CD68) demonstrated a worse DFS in TN. Higher H-score (CD4) and H-score (CD8) represented a better DFS in TS (**Figure 5**). Kaplan–Meier curves illustrated the associations between the infiltrating proportion rates of immune biomarkers (high *vs*. low) within the TN and TS and DFS (**Figure 6**). The prognostic effects of cell density and number of immune cells were available in **Supplementary Table S6** and **Supplementary Table S7**. The distinction of the infiltration levels of several immune biomarkers was statistically significant across T stage and N stage (**Supplementary Figure S3**)—for instance, the infiltration levels of intratumoral CD4-positive T cells and CD8-positive T cells were lowest in T4 stage, while the levels of intrastromal PD-L1-positive cells were highest in T4 stage and N1 stage. Moreover, as mentioned above, the immune-exempted subtype had the lowest levels of infiltrating immune cells other than Treg and neutrophils and had a more advanced T stage and clinical stage, suggesting that stage may also play a pivotal role in the prediction of clinical response.

### Prognoses of Immune-Related Risk Score

**Figure 7:**
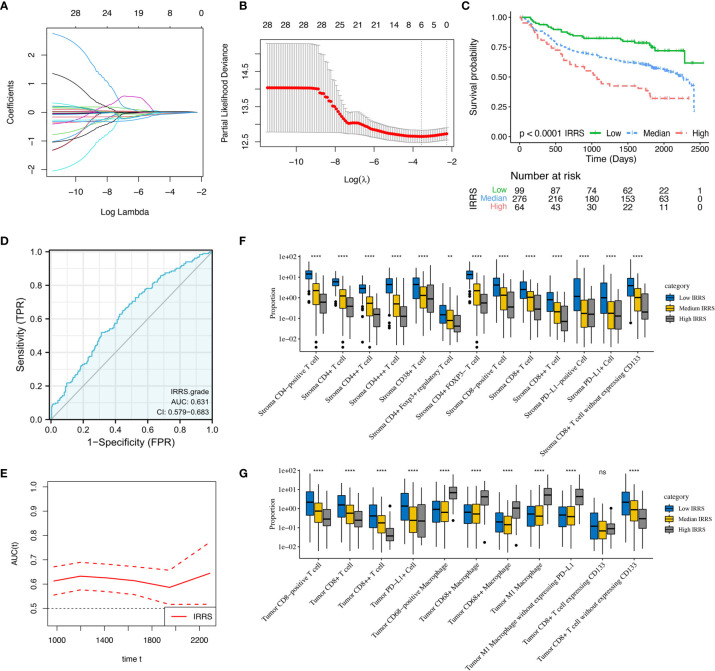
Construction and validation of immune-related risk score (IRRS) in the training cohort. **(A)** Least absolute shrinkage and selection operator (LASSO) coefficient profiles of 28 selected immune cell biomarkers in the 10-fold cross-validation. **(B)** Partial likelihood deviance revealed by the LASSO regression model in the 10-fold cross-validation. **(C)** Kaplan–Meier curve estimates the differences of disease-free survival, divided by three IRRS subtypes (low vs. median vs. high). **(D)** Receiver operating characteristic curves and area under curve (AUC) values indicated the accuracy of the IRRS model. **(E)** Time-dependent AUC curve estimating the prognostic performance of IRRS. **(F, G)** Box plots present the infiltration disparities of three IRRS subgroups in tumor nest and tumor stroma. **P < 0.01; ****P < 0.0001; ns, nonsignificant.

Intratumoral CD68-positive macrophages, M1 macrophages, and intrastromal CD4+ cells, CD4+FOXP3- cells, CD8+ cells, and PD-L1+ cells were found to be the robust prognostic biomarkers through LASSO (minimized lambda = 0.0281), and their regression coefficients derived for multivariable Cox proportional hazards regression analysis were 1.033, 1.035, 0.922, 0.968, 0.875, and 0.925, respectively (**Figures 7A, B**). Hence, the following formula was utilized to calculate the IRRS for each patient: IRRS = (intratumoral - %CD68-positive) * ln (1.033) + (intratumoral - % M1 macrophages) * ln (1.035) + (intrastromal - %CD4+) * ln (0.922) + (intrastromal - % CD4+FOXP3- cells) * ln (0.968) + (intrastromal - %CD8+) * ln (0.875) + (intrastromal - %PD-L1+) * ln (0.925). Multivariable Cox regression analysis showed that IRRS was significantly associated with DFS in the training cohort (*p* < 0.001). We further stratified all patients into high IRRS, median IRRS, and low-IRRS subtypes using -0.01 and -0.86 as the optimal cutoff values. As illustrated in **Table 1**, the low IRRS subtype had the most favorable DFS, whereas the high IRRS subtype showed the worst (HR 2.63, 95%CI 1.86–3.71, *P* < 0.001), suggesting a relatively great ability for risk stratification (**Figure 7C**). Moreover, patients with median IRRS also had longer DFS than patients with high IRRS (HR 0.34, 95%CI 0.23–0.50, *P <*0.001). The AUC under the ROC curve evaluating the prognostic accuracy of IRRS model was 0.631 (95%CI 0.579–0.683), 0.613 (95%CI 0.568–0.657), and 0.555 (95%CI 0.472–672) of the training, entire, and testing cohort, respectively (**Figure 7D** and **Supplementary Figures S4**, **S5**). The prognostic performance of IRRS was also assessed using time-dependent AUC curve, of which the training cohort indicated that the AUC fluctuated between 0.6 and 0.7, while AUC in the entire cohort gradually reached 0.9 after 2,500 days, implying that the IRRS model had a higher predictive effect on long-term risk in relapse (**Figure 7E** and **Supplementary Figure S4**). Moreover, with the increment of IRRS, the infiltrating levels of CD4-positive cells, CD8-positive cells, and CD38-positive cells decreased gradually, while the levels of macrophages increased gradually (**Figures 7F, G** **)**. Similar trends that patients in the high IRRS subgroup had a significantly worse DFS than the median IRRS and low IRRS subgroups, were observed in the testing cohort (1,031 *vs*. 1,710 *vs*. 1,792 days, *P* = 0.001) and the entire cohort (985 days *vs*. 1,678 days *vs*. 1,725 days, *P* < 0.001). The IRRS also showed a potential ability for risk stratification (high *vs*. median *vs*. low) and prediction of 5-year DFS rates (43.1 *vs*. 37.9 *vs*. 23.2%, *P* < 0.001) in the entire cohort.

**Table 1:** Construction and validation of immune-related risk score for predicting the disease-free survival of non-small cell lung cancer.

Variables	Univariate analysis		Training cohort		Testing cohort		Entire cohort	
			Multivariate analysis		Multivariate analysis		Multivariate analysis	
	HR (95%CI)	*P*-value	HR (95%CI)	*P*-value	HR (95%CI)	*P*-value	HR (95%CI)	*P*-value
Age	1.01 (1.00, 1.02)	0.055	1.01 (1.00, 1.03)	0.091	1.01 (0.99, 1.03)	0.431	1.01 (1.00, 1.03)	0.027
Sex								
Male	Ref.		Ref.		Ref.		Ref.	
Female	0.70 (0.54, 0.90)	0.005	0.72 (0.51, 1.00)	0.049	0.81 (0.47, 1.38)	0.437	0.74 (0.56, 0.96)	0.026
Tstage								
T1	Ref.		Ref.		Ref.		Ref.	
T2	1.05 (0.77, 1.41)	0.770	0.70 (0.48, 1.03)	0.073	1.24 (0.67, 2.28)	0.498	0.86 (0.64, 1.18)	0.354
T3	1.64 (1.16, 2.32)	0.005	1.63 (1.06, 2.52)	0.025	1.00 (0.47, 2.13)	0.991	1.18 (0.82, 1.70)	0.364
T4	2.85 (1.93, 4.22)	<0.001	2.30 (1.35, 3.94)	0.002	3.02 (1.38, 6.63)	0.006	2.27 (1.50, 3.44)	<0.001
Nstage								
N0	Ref.		Ref.		Ref.		Ref.	
N1	2.85 (2.03, 4.00)	<0.001	3.52 (2.27, 5.46)	<0.001	1.76 (0.83, 3.73)	0.138	2.91 (2.03, 4.18)	<0.001
N2	3.40 (2.56, 4.52)	<0.001	3.05 (2.11, 4.42)	<0.001	2.40 (1.35, 4.25)	0.003	3.00 (2.23, 4.03)	<0.001
Visceral pleural invasion								
PL0	Ref.		Ref.		Ref.		Ref.	
PL1	1.45 (1.11, 1.90)	0.006	1.14 (0.79, 1.65)	0.478	1.16 (0.69, 1.97)	0.579	1.12 (0.84, 1.50)	0.442
PL2	1.26 (0.76, 2.11)	0.370	1.58 (0.81, 3.06)	0.177	0.54 (0.16, 1.88)	0.334	1.21 (0.69, 2.12)	0.498
Vascular tumor emboli								
No	Ref.		Ref.		Ref.		Ref.	
Yes	2.05 (1.58, 2.65)	<0.001	1.43 (1.07, 1.92)	0.017	1.49 (0.86, 2.56)	0.152	1.36 (1.02, 1.79)	0.033
Resected lymph nodes	1.00 (0.98,1.01)	0.613	0.98 (0.97, 1.00)	0.015	0.98 (0.96, 1.01)	0.192	0.98 (0.97, 1.00)	0.012
Immune-related risk score								
Low	Ref.		Ref.		Ref.		Ref.	
Median	1.65 (1.21, 2.24)	0.001	1.94 (1.22, 3.08)	0.005	1.81 (1.02, 3.21)	0.044	1.62 (1.18, 2.23)	0.003
High	2.63 (1.86, 3.71)	<0.001	3.56 (2.06, 6.15)	<0.001	2.77 (1.59, 4.80)	<0.001	2.98 (2.02, 4.40)	<0.001

### Relationships Between Histological Staining and Multiplex Immunofluorescence

Technically, histological aspects are not related to a specific pathway of MIF. The fundamental principle of MIF is that diverse biomarkers (*i*.*e*., protein) can be stained by specific antibodies labeled with distinct fluorophores singly (47). Moreover, given that the immunofluorescence fluorophores have a dynamic scope, the IF staining of tissue is capable to characterize cells phenotypically (*e*.*g*., different functional states or different development phases of cells), which cannot be achieved by histological slides. Nevertheless, the cell and tissue segmentation were conducted based on the morphologies of histological and multispectral images, and hence they are complementary in this respect.

## Discussion

We presented a MIF method for the simultaneous identification of colocalized biomarkers in immune cell phenotyping in TIME. This is the first study to highlight the comprehensive characteristics and clinical significance of *in situ* immune cells from resected NSCLC using a large cohort. Firstly, we identified three robust immune subtypes through unsupervised consensus clustering method, including immune-activated, immune-exempted, and immune-defected. Each of the immune subtypes was correlated with distinct infiltrating immune cell levels and accordingly indicating significantly different prognosis. After that, we presented an IRRS model utilizing multivariable Cox regression and LASSO regression analyses, clearly demonstrating the potential ability for risk stratification and prognosis prediction for DFS.

Our findings have several strengths and the following important aspects which differ from previous studies. This is the first study that investigated the density, proportion, number, and H-score for each type of immune cells in both tumor and paratumor stroma. We implemented the MIF test for 66 kinds of immune biomarkers from stage IA to IIIB NSCLC in a large cohort of 681 patients, avoiding shortcomings like a homogeneous cohort (a particular clinical stage), low statistical power, and wide variation. Moreover, except for using established biomarkers, we additionally tested other immune biomarkers which were rarely reported like CD8+CD133+ and CD4+CD38+ and further analyzed their functional state by the presence of PD-L1, reflecting multifarious immunological processes. In addition, traditional prognostic biomarkers were usually developed by an individual-based model which requires the information of clinical outcomes to be known in advance, namely, “supervised”. On the contrary, we utilized the unsupervised consensus clustering approach based on the levels of immune biomarker profiles to reveal the intricate and intrinsic structure of TIME, maximizing the homogeneity of immune components within the same cluster and the heterogeneity among different clusters. Finally, we designed an IRRS model based on quantitative evaluation of infiltrating immune cells specific to the constitution of TIME rather than non-specific gene signatures that were generally used in previous studies. The candidate factors were selected in a rigorous method based on multivariate Cox regression and LASSO regression analyses, enhancing the statistical power.

A study from Chen et al., which focused on head and neck cancer, presented three immune subtypes, namely, non-immune, exhausted, and active, respectively (48). Similarly, the immune-activated class accounted for the largest proportion of our patients. The immune-exempted subtype was consistent with Chen’s defined non-immune class which showed significantly lower infiltrating levels of lymphocytes and a more advanced T stage. The immune-defected class, in which macrophages tended to exhibit pro-tumor activity under the impact of CSC, however, has not been reported yet. Therefore, our findings recapitulated the immune classes and complemented previous studies. It is noteworthy that intracluster heterogeneity was also observed in our analysis, suggesting that novel methods for clustering should be developed in the future.

The impact of immune profiles in TIME on the survival of patients has been well described across cancer types. The immune-activated subtype in our study showed the highest infiltration of immune effectors like CD4+ T cells, CD20+ B cells, and CD8+ T cells without expressing CD133, and accordingly, patients in this class had the longest DFS. On the contrary, immune-defected tumors had a mass of CSC and macrophages, indicating the worst outcome. It is noteworthy that macrophages primarily originate from the bone marrow and polarize by tumor-derived signals (49). Two major lineages, including M1 and M2, of polarization have been well described. Generally, M1 macrophages mainly display antitumoral functions by secreting cytokines for T cell activation, while M2 macrophages are perceived as pro-tumor effectors through angiogenesis and the chemotactic function of Tregs (50). Consistent with these findings, M2 macrophages without expressing PD-L1 were enriched in the immune-defected subtype. However, we observed that a higher infiltration of M1 macrophages was also associated with shorter DFS, and so the specific function of macrophages still needs to be evaluated synthetically. Immune-exempted tumors were dominated by neutrophils and Tregs, which were critical for creating an immunosuppressive microenvironment through TGF-β and IL-10 signaling, and accordingly, patients were in a more advanced clinical stage. Importantly, the role of neutrophils in the development of LC was still divergent so far. Evgeniy B. and colleagues have previously reported that neutrophils could stimulate T cell responses in early-stage LC by increasing IFN-γ production (44). In comparison, patients in the immune-exempted subtype were mainly enriched in advanced stage, and so our findings may suggest that neutrophils tend to exhibit a pro-tumor rather than an anti-tumor effect in advanced LC.

Our findings may offer a reference for designing rational combination immunotherapy strategies—for instance, patients in the immune-activated class may benefit from single-agent ICB, reinforcing the preexisting anti-tumor responses and further extending their survival. As for the immune-exempted and immune-defected subtypes, ICB alone may not be sufficient, considering the presence of immunosuppressive mechanisms. In this regard, TGF-β inhibition (NCT02423343 and NCT04064190 are ongoing trials), radiotherapy, or chemotherapy plus ICB can be utilized to change a non-inflamed malignancy into an inflamed one and further stimulate the dampened anti-tumor immunity (51). Novel approaches for these two subtypes, like transferring of neoantigen-reactive T cells and NK cells which can enhance anti-tumor immunological effects, are under active investigation. For patients with intracluster heterogeneity, therapeutic selections should depend on the specific TIME and usage of targeting carcinoma-associated fibroblast therapies (52) or anti-angiogenic therapies (53), plus ICB may work. It is noteworthy that, given only a single method rather than combined methods with quantitative PCR and fluorescence-activated cell sorting analysis that we used, researchers should interpret our results with caution. In summary, further studies with multiomics data are in an urgent need to detect the exact molecular and cellular mechanisms responsible for immune inactivity for curating novel combination strategies.

Our study also provided evidence for complicated correlations among immune cells, like Tregs and neutrophils, CD20-positive B cells and CD4+CD38+ T cells, and CSC and macrophages, implying that the chemotactic function may contribute to the formation and evolution of TIME. Moreover, we found that the prognostic significance of each biomarker varied from low-fluorescence intensity (+) to high-fluorescence intensity (+++), implying that the fluorescence intensity may represent the different functional states or different development phases of cells. Interestingly, we observed that the prognosis effects of cells with median fluorescence intensity (++) were mostly more significant than that with low fluorescence intensity (+) or high fluorescence intensity (+++) ones, suggesting that this kind of cells was likely to be most functional, and further investigation is warranted.

Several limitations existed in our study. Firstly, although we investigated the prognostic significance for as many kinds of immune biomarkers as possible, the biological mechanisms behind them were unclear, and further experimental studies are warranted. Secondly, our patient cohort did not include stage IV samples, and the proportion of stage IIIB samples was limited as well (0.5%). Therefore, further studies should pay more attention to covering advanced-stage NSCLC. In addition, we could not make an external validation of IRRS model. Hence, the generalization of our findings needs to be confirmed by more studies. Finally, due to the lack of treatment information, we could not assess the value of IRRS in predicting treatment response.

In summary, we comprehensively demonstrated the immune landscape of NSCLC through MIF analysis and further identified three robust immune subtypes, which may help identify the ideal candidates and tailor rational immunotherapeutic strategies. We also revealed the prognostic significance of 66 kinds of immune biomarkers and subsequently constructed an IRRS model for predicting the DFS of patients, attributing to the risk stratification and prognosis prediction for DFS. Future studies with a larger sample size and a better design are warranted for our deeper understanding of TIME.

## Data Availability Statement

The raw data supporting the conclusions of this article will be made available by the authors, without undue reservation.

## Author Contributions

HP, XW, and WL contributed to conception and design. HP, XW, and RZ contributed to administrative support. TY, XC, JL, YW, YA, JC, YL, MH, and CL contributed to the provision of study materials or patients. HZ and YC contributed to the collection and assembly of data. HP and XW contributed to data analysis and interpretation. All authors contributed to the article and approved the submitted version.

## Funding

This work was supported by the China National Science Foundation (grant number 81871893), the Key Project of Guangzhou Scientific Research Project (grant number 201804020030), and the Cultivation of Guangdong College Students’ Scientific and Technological Innovation (“Climbing Program” Special Funds; grant numbers pdjh2020a0480 and pdjh2021a0407).

## Conflict of Interest

TY, HZ, and YC are employed by Genecast Biotechnology Co., Ltd.

The remaining authors declare that the research was conducted in the absence of any commercial or financial relationships that could be construed as a potential conflict of interest.

## Publisher’s Note

All claims expressed in this article are solely those of the authors and do not necessarily represent those of their affiliated organizations, or those of the publisher, the editors and the reviewers. Any product that may be evaluated in this article, or claim that may be made by its manufacturer, is not guaranteed or endorsed by the publisher.

## Supplementary Material

Supplementary Figure S1 Positive and negative control used in the multiplex immunofluorescence (MIF) approach. **(A)** MIF image of lung tumor, **(B)** negative control MIF image from normal human tonsil tissue, and **(C)** positive control MIF image from normal human tonsil tissue.

Supplementary Figure S2 Identification of differences in the spatial distribution of immune cells within tumor nest and tumor stroma using Kruskal–Wallis test. **P* < 0.05, ***P* < 0.01, ****P* < 0.001. ns, no significant difference.

Supplementary Figure S3 Several immune biomarkers significantly associated with disease-free survival (*p* < 0.05) are selected, and *T*-test is performed in the identification of infiltration distinction across clinical stage, T stage, and N stage in the tumor nest **(A)** and tumor stroma **(B)**.

Supplementary Figure S4 Kaplan–Meier curves of three immune-related risk score (IRRS) subgroups of the entire cohort **(A)**. Receiver operating characteristic curve **(B)** and time-dependent area under curve **(C)** estimating the prognostic performance of IRRS. Box plots present the infiltration disparities of three IRRS subgroups in the tumor nest **(D)** and tumor stroma **(E)**.

Supplementary Figure S5 Kaplan–Meier curves of three immune-related risk score (IRRS) subgroups of the testing cohort **(A)**. Receiver operating characteristic curve **(B)** and time-dependent area under curve **(C)** estimating the prognostic performance of IRRS. Box plots present the infiltration disparities of three IRRS subgroups in the tumor nest **(D)** and tumor stroma **(E)**.

## Abbreviations

AUC, area under curve; CD, cluster of differentiation; CDF, cumulative distribution function; CI, confidence interval; CTLA-4, cytotoxic T-lymphocyte antigen 4; DCA, decision curve analysis; DFS, disease-free survival; FOXP3, Forkhead box P3; HR, hazard ratio; H-score, histochemical score; ICB, immune checkpoint blockade; IFN-γ, interferon-γ; IL, interleukin; IRRS, immune-related risk score; K–W test, Kruskal–Wallis test; LASSO, least absolute shrinkage and selection operator; MMPs, matrix metallopeptidases; NCCN, The National Comprehensive Cancer Network; NSCLC, non-small cell lung cancer; OS, overall survival; PCA, principal component analysis; PD-L1, programmed death-ligand 1; SCLC, small cell lung cancer; TAMs, tumor-associated macrophages; TGF-β, transforming growth factor-β; TH, T-helper cell; TIL-Bs, tumor-infiltrating B cells; TILs, tumor-infiltrating lymphocytes; TIME, tumor immune microenvironment; TLS, tertiary lymphoid structure; Treg, regulatory T cell; TSA, tyramine signal amplification.
