# Supplementary figures and images for: Profiling Tumor Immune Microenvironment of Non-Small Cell Lung Cancer Using Multiplex Immunofluorescence
Source: Front Immunol. 2021 Nov 4;12:750046. doi: 10.3389/fimmu.2021.750046 (PMC8600321; doi:10.3389/fimmu.2021.750046)

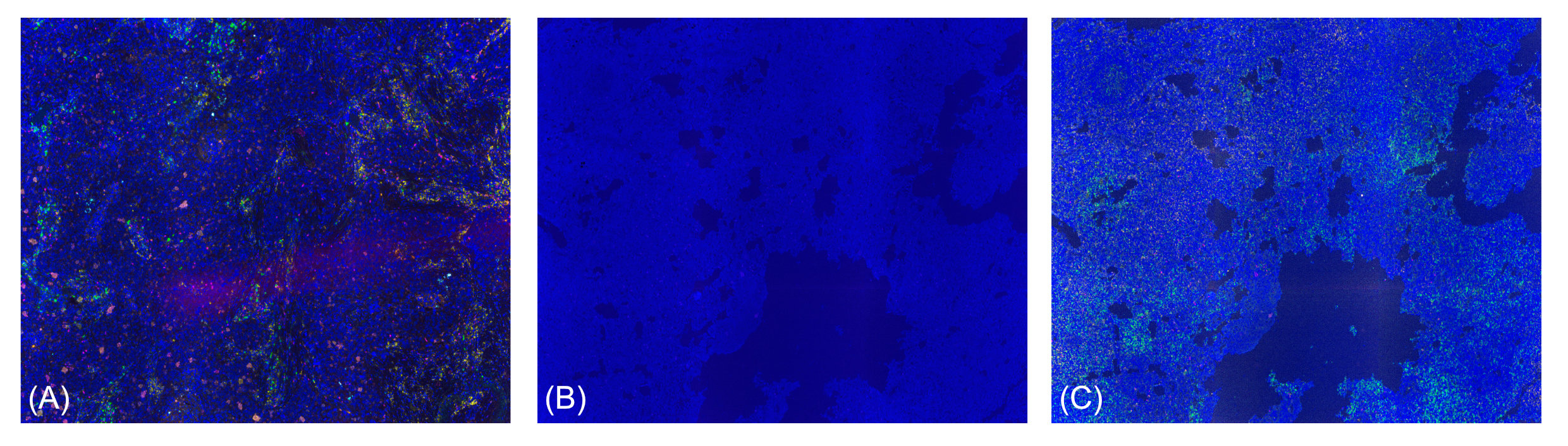

Supplement: Supplementary Figure S1 — Positive and negative control used in the multiplex immunofluorescence (MIF) approach. (A) MIF image of lung tumor, (B) negative control MIF image from normal human tonsil tissue, and (C) positive control MIF image from normal human tonsil tissue. [file Image_1.jpeg]

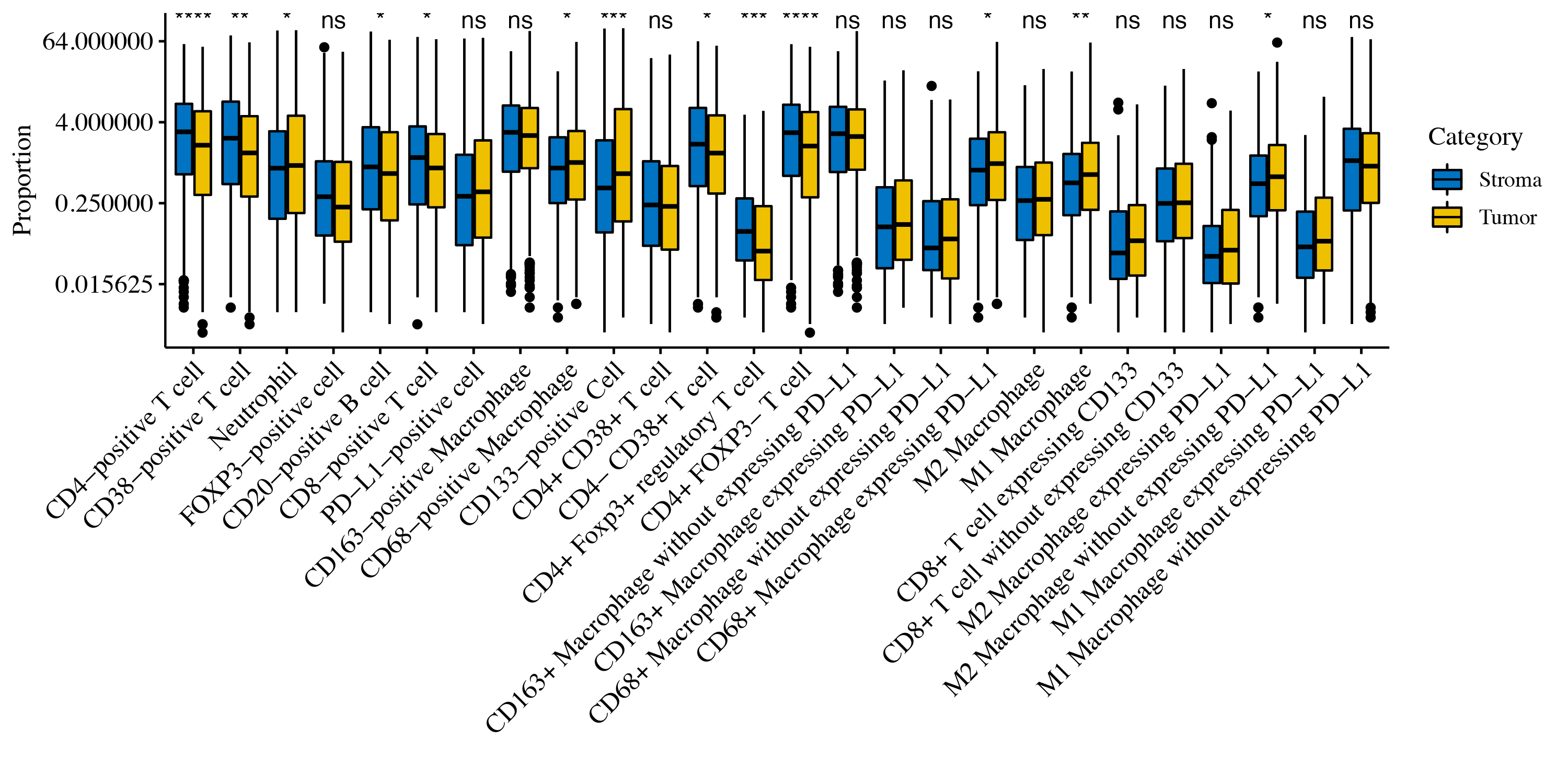

Supplement: Supplementary Figure S2 — Identification of differences in the spatial distribution of immune cells within tumor nest and tumor stroma using Kruskal–Wallis test. *P < 0.05, **P < 0.01, ***P < 0.001. ns, no significant difference. [file Image_2.jpeg]

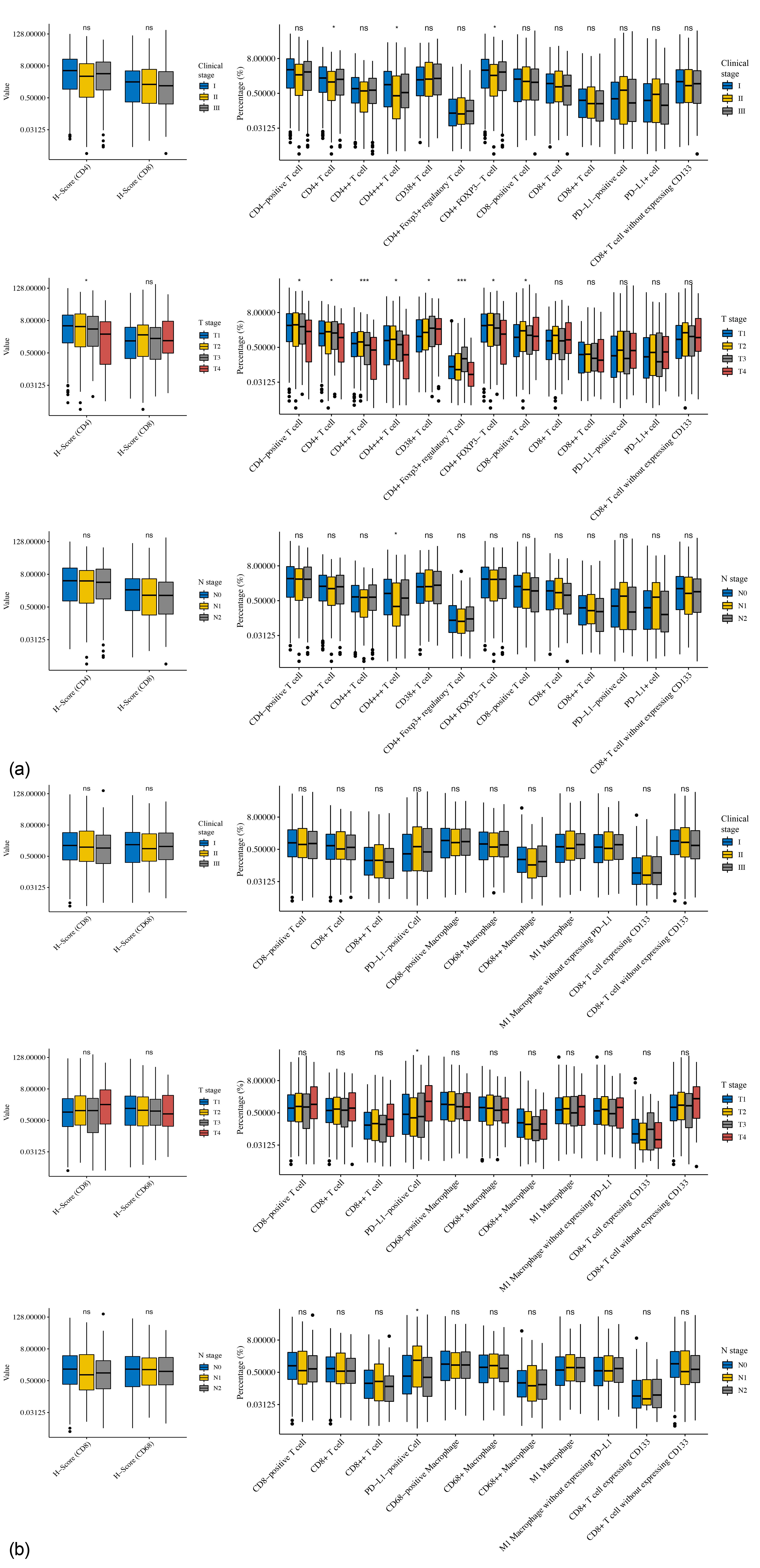

Supplement: Supplementary Figure S3 — Several immune biomarkers significantly associated with disease-free survival (p < 0.05) are selected, and T-test is performed in the identification of infiltration distinction across clinical stage, T stage, and N stage in the tumor nest (A) and tumor stroma (B). [file Image_3.jpeg]

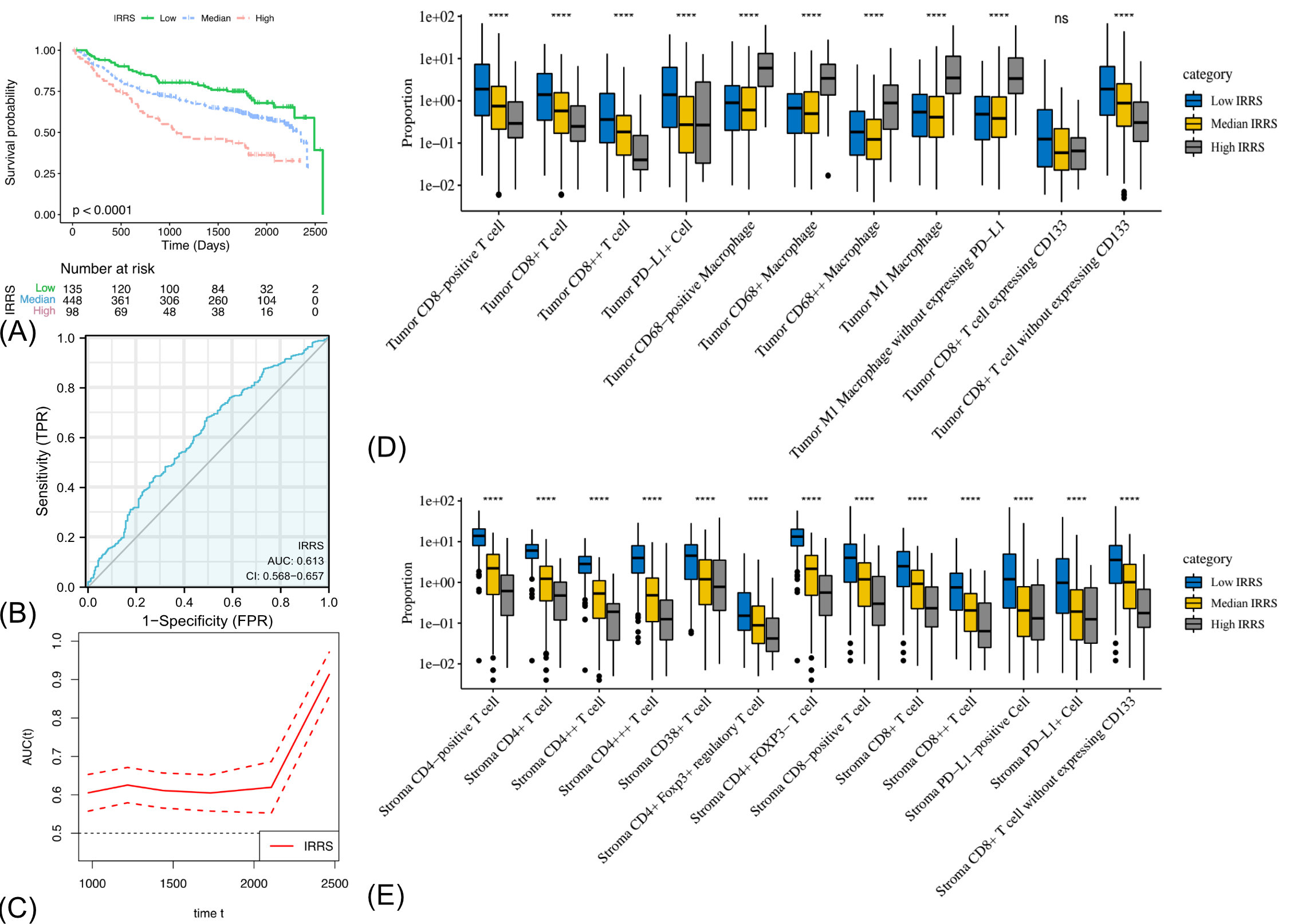

Supplement: Supplementary Figure S4 — Kaplan–Meier curves of three immune-related risk score (IRRS) subgroups of the entire cohort (A). Receiver operating characteristic curve (B) and time-dependent area under curve (C) estimating the prognostic performance of IRRS. Box plots present the infiltration disparities of three IRRS subgroups in the tumor nest (D) and tumor stroma (E). [file Image_4.jpeg]

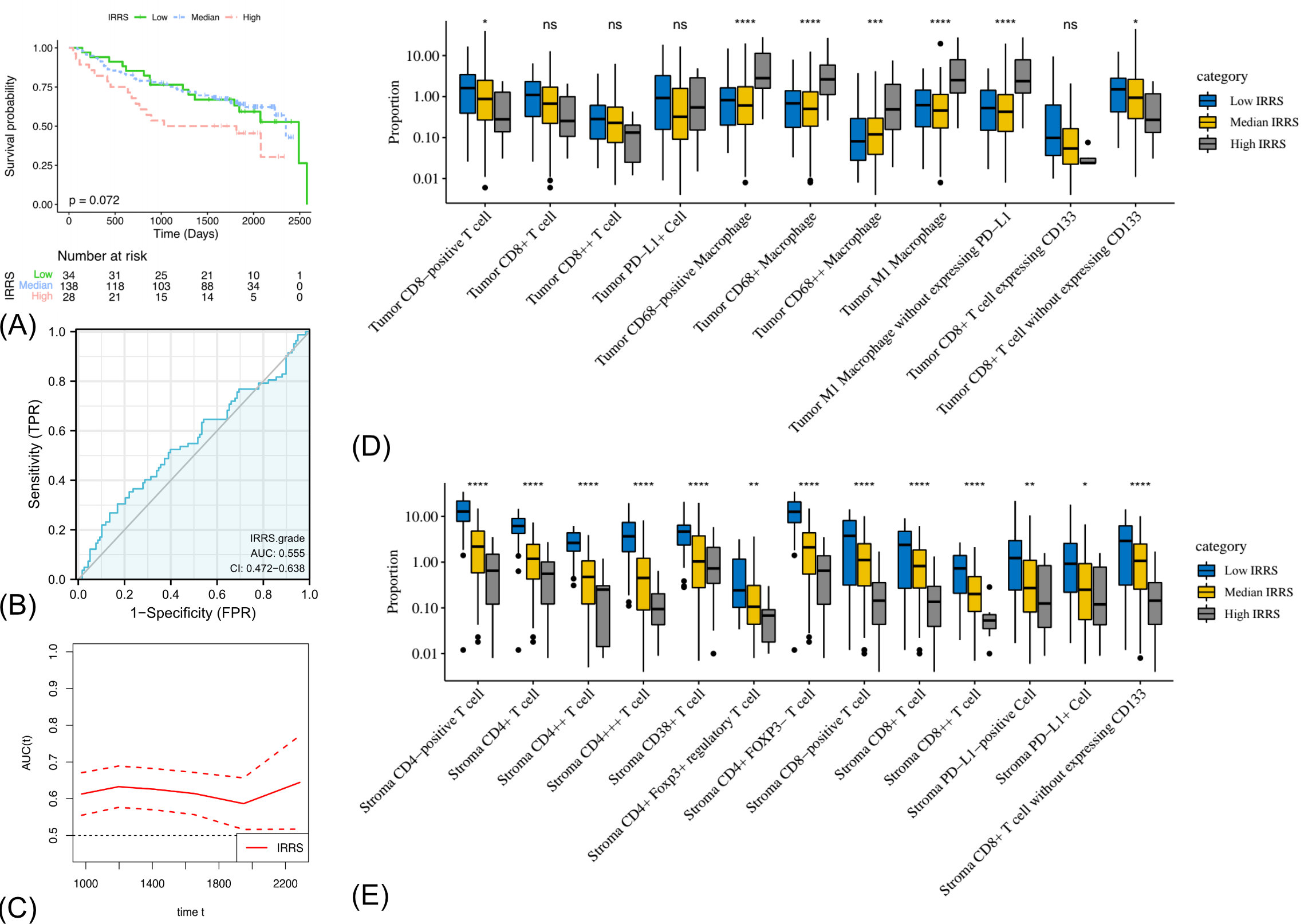

Supplement: Supplementary Figure S5 — Kaplan–Meier curves of three immune-related risk score (IRRS) subgroups of the testing cohort (A). Receiver operating characteristic curve (B) and time-dependent area under curve (C) estimating the prognostic performance of IRRS. Box plots present the infiltration disparities of three IRRS subgroups in the tumor nest (D) and tumor stroma (E). [file Image_5.jpeg]
